# Using Solubility Parameters to Model More Environmentally Friendly Solvent Blends for Organic Solar Cell Active Layers

**DOI:** 10.3390/ma12233889

**Published:** 2019-11-25

**Authors:** Ishita Jalan, Lisa Lundin, Jan van Stam

**Affiliations:** Department of Engineering and Chemical Sciences, Karlstad University, SE-651 88 Karlstad, Sweden; Ishita.Jalan@kau.se (I.J.); lisa.lundin76@gmail.com (L.L.)

**Keywords:** organic solar cells, Hansen solubility parameters, solvent blends, solubility

## Abstract

To facilitate industrial applications, as well as for environmental and health purposes, there is a need to find less hazardous solvents for processing the photoactive layer of organic solar cells. As there are vast amounts of possibilities to combine organic solvents and solutes, it is of high importance to find paths to discriminate among the solution chemistry possibilities on a theoretical basis. Using Hansen solubility parameters (HSP) offers such a path. We report on some examples of solvent blends that have been found by modelling HSP for an electron donor polymer (TQ1) and an electron acceptor polymer (N2200) to match solvent blends of less hazardous solvents than those commonly used. After the theoretical screening procedure, solubility tests were performed to determine the HSP parameters relevant for the TQ1:N2200 pair in the calculated solvent blends. Finally, thin solid films were prepared by spin-coating from the solvent blends that turned out to be good solvents to the donor-acceptor pair. Our results show that the blend film morphology prepared in this way is similar to those obtained from chloroform solutions.

## 1. Introduction

An advance in the field of organic solar cells (OSCs) is of increasing interest, both from a fundamental and an applied point of view, as OSCs offer broad opportunities to produce electricity in a sustainable way as well as showing multiple technical benefits, e.g., solution processability, flexibility, and light-weight [1,2,3,4,5,6]. The commercialization of OSC technology will depend on three key factors: Device efficiency, lifetime, and cost [7]. 

An OSC device is schematically build up by a core photoactive layer, or active layer, between two electrodes. There is often an electron transport layer and a hole transport layer between the active layer and the electrodes, respectively. The active layer is commonly prepared from a solution of at least two solutes; the electron donor and the electron acceptor. The donor is regularly a polymer, while the acceptor can be, for instance, a fullerene derivative, a small molecule, or another polymer. Recently, the interest is shifted from fullerene-based acceptors towards non-fullerene donors, in order to prevent photochemical degradation; a well-known problem of many fullerene derivatives [8].

When preparing the active layer, the donor-acceptor solution is deposited as a thin liquid film upon a substrate—there are different methods for this process—and, subsequently, the solvent evaporates to leave a solid thin blend film with a typical thickness of about 100 nm on the substrate. During the drying process, the concentration of the solutes increases, and eventually a phase-separation of the dissolved species occurs. Due to the fast evaporation of the solvent, this phase-separation will be only partial, creating a structure in the thin blend film. This structure, the film morphology, is decisive for the OSC performance [9,10,11,12] There is, hence, attention paid to increase the understanding of the drying process and how to control the process to yield an optimal morphology for a given donor-acceptor pair. Of vital importance are the pairwise interactions between the solutes and the solvent, i.e., the solution chemistry of the system.

The solvents commonly used, e.g., halogenated hydrocarbons, often aromatic, work well in a laboratory. For scaling-up, however, it is necessary to find alternatives that are less harmful to environment and health. The solubility of the molecular components depends on how well the solutes and solvents used match each other. As there is an excess of possibilities to combine organic solvents and solutes, we need ways to discriminate between possible solvents for a chosen pair of donor and acceptor. The use of Hansen´s solubility parameters (HSP) [13,14,15,16,17] is such a feasible way, appealing in its straightforwardness, especially when finding suitable solvent blends. HSP is theoretically based on thermodynamics and regular solution theories and relies on how the dispersion forces, polar forces, and hydrogen bonding forces (denoted as δD, δP, and δH, respectively) influence the interactions between solvent and solutes and hence the solubility. This model has successfully been applied to several OSC systems [4,16,17,18,19,20,21].

In this report, we show how HSP can be used to find solvent blends for an all-polymer donor-acceptor pair, applicable in topical work on OSCs. We have chosen a polymer-polymer system to examine the solution chemistry challenge of dissolving two polymers in the same solvent blend. The calculations were performed with the program *Hansen Solubility Parameters in Practice* (HSPiP) [22]. The morphology of the films prepared from alternative solvents are compared with that of films from a more commonly used solvent, i.e., chloroform. To characterize the blend film morphology, atomic force microscopy (AFM) was used. 

## 2. Materials and Methods 

### 2.1. Materials

**Figure 1:**
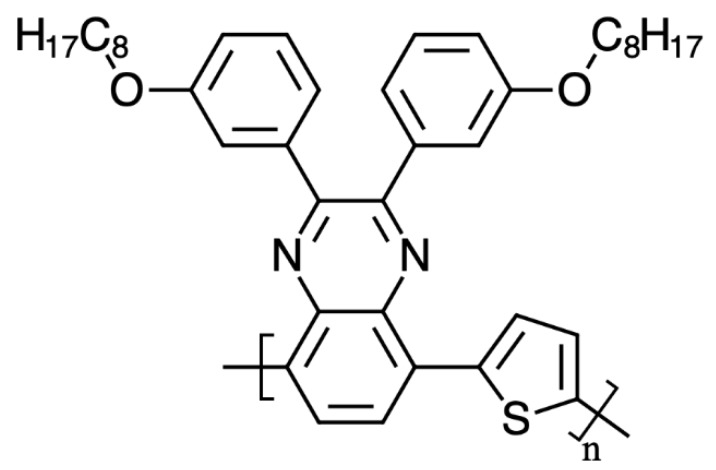
The structure of the donor polymer TQ1.

**Figure 2:**
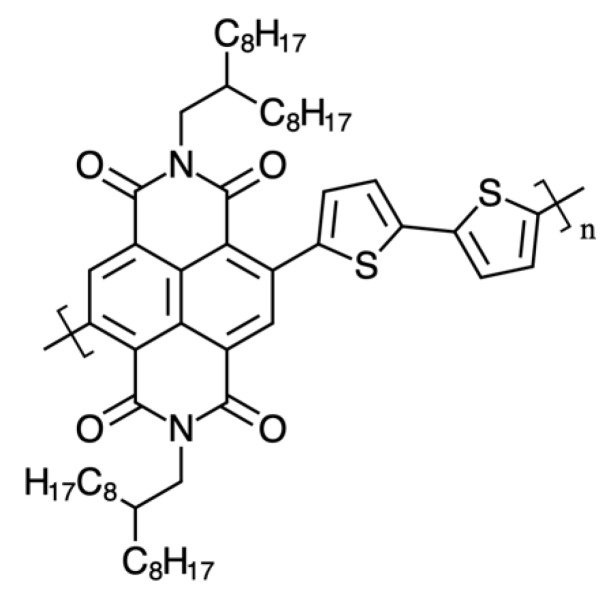
The structure of the acceptor polymer N2200.

The donor polymer, poly[2,3-bis-(3-octyloxyphenyl) quinoxaline-5,8-diyl-alt-thiophene-2,5-diyl], Figure 1 (TQ1), was purchased from Lumtec, with a number averaged molecular weight, M_n_, of 31,800 and a polydispersity index (PDI) of 3.11. The acceptor polymer, poly{[N,N′-bis(2-octyldodecyl)-naphthalene-1,4,5,8-bis(dicarboximide)-2,6-diyl]-alt-5,5′-(2,2′-bithiophene)}, Figure 2 (N2200 or P[NDI2OD-T2]), was purchased from Ossila, with an M_n_ of 150,500 and a PDI of 1.9. The solvents used are summarized in Table 1. The solvents were chosen to be less hazardous to environment and health, as compared to the solvents often used to prepare OSC. The *GSK Solvent Selection Guide 2009* can be used as a good starting point in finding alternatives for the halogenated solvents [23]. This guide was used to discriminate solvents in this contribution.

### 2.2. Solubility Tests of N2200 and TQ1—Determining the HSP by Using the HSPiP Program

In order to determine the HSP values for N2200 and TQ1, solubility tests were performed. In principle, it is possible to perform the solubility test with as few as ten solvents. As the precision of the determined HSP values becomes better if a larger number of solvents is used, 32 different solvents were used in the solubility test. A compilation of the solvents, with their HSP values, is given in Table 2. For N2200, the initial solubility test was performed with a concentration of 1.0 mg per 1.0 ml of solvent in a transparent glass vial. The vial was heated to 50 °C and checked after 1, 24, 48, 72, and 96 hours.

For those solvents that dissolved the polymer under these conditions, a second test was performed similarly, but with a concentration of 5.0 mg/ml. Finally, a third test was performed with the solvents that dissolved the higher N2200 concentration, now with a concentration of 10.0 mg/ml.

**Figure 3:**
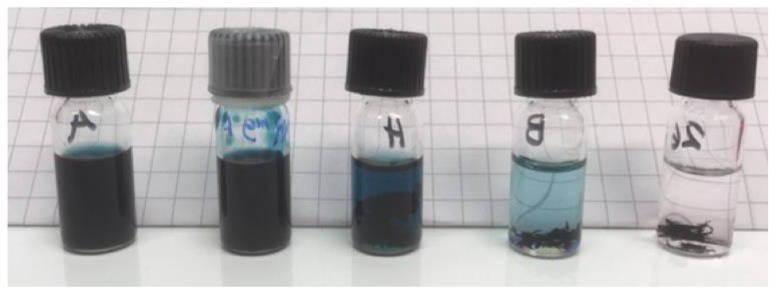
An illustrative example of how different solubilisation behaviors were scored. The scores are, from left to the right, 1, 2, 3, 4, and 0.

The results of the solubility tests were scored as (see Figure 3 for an illustrative example):

1 = Dissolved. Deep dark blue color.

2 = Some undissolved polymer. Quite dark color. 

3 = Polymer is only partially dissolved. Light blue color.

4 = Very little polymer is dissolved. Pale-light blue color. 

0 = Clear solvent with no dissolved polymer.

These scores were put into the HSPiP program, and the HSP values of N2200 were calculated. The same procedure was used for TQ1, but only with the highest concentration, i.e., 10 mg/ml. It should be mentioned that the HSP values depend on the solubility threshold determined by the researcher and the experimental conditions. The HSP values of the solute and the values of the solvents are used to calculate possible good solvent blends by the HSPiP module *Optimizer* [13,22]. The program calculates two distances in Hansen space (with the three axes δD, δP, δH). The first distance, R_0_, is the radius of the solubility sphere determined by the solubility tests, with the good solvents inside the sphere and the bad solvents outside. The second distance, R_a_, is the distance in Hansen space between solute(s) and the solvent/solvent blend. R_a_ is calculated for the two molecules by:

(1) $$R_{a}^{2}=4{\left(\delta D_{2}-\delta D_{1}\right)}^{2}+{\left(\delta P_{2}-\delta P_{1}\right)}^{2}+{\left(\delta H_{2}-\delta H_{1}\right)}^{2}$$

The fit of R_a_ can be judged by the core values reported by the HSPiP program. They are given in the form ±[ δD, δP, δH] and should preferably be less than 0.5 for each component.

The fitted distances are used to calculate the relative energy distance (RED) value (RED = R_a_/R_0_). In this model, a RED ≤1 is indicative for a situation where the solute will be dissolved by the solvent, while RED >1 leads to an undissolved solute. The blends with RED ≤1 were subsequently used for a new series of solubility measurements. A more elaborated discussion on the theoretical part of the HSP model is given in [13,15], and tutorials can be found in [22].

Finally, the blends that showed to be good solvents for the TQ1-N2200 donor-acceptor pair in the solubility tests were used to prepare thin blend films by spin-coating the solution on a glass substrate. These films were characterized by AFM and compared to films prepared from solutions with chloroform as a solvent.

### 2.3. Active Layer Morphology Determined by AFM

Atomic force microscopy images of TQ1, N2200, and the blend thin films were obtained with Nanoscope Multimode 8 (Bruker, USA) in PeakForce Tapping mode (ScanAsyst), controlled by Nanoscope 9.2 software, using a Si tip in air.

The blend films for AFM characterization were spin-coated on glass substrates at 1000 rpm from solutions with at total solute concentration of 10 mg/ml. After coating, the films were heated to 250 °C for 1 minute to ensure complete solvent evaporation, followed by thermal annealing at 120 °C for 10 minutes. When reference blend films were spun from chloroform, no pre-annealing was necessary, due to the high vapor pressure of chloroform. The weight ratio of TQ1:N2200 was 1:1 and 2:1.

## 3. Results and Discussion

The HSP values for N2200 were determined from the solubility measurements. Together with earlier reported HSP values for TQ1 [4], possible solvent blends of non-halogenated solvents were calculated by modelling in the HSPiP program. These solvent blends were used for solubility experiments of TQ1:N2200 blends and from successful solvent blends; thin blend films were prepared by spin-coating. The films were characterized by AFM and their morphology compared with films similarly prepared from chloroform.

### 3.1. HSP for N2200 and TQ1-N2200 Double Sphere

The solvents used for determining the HSP values for N2200 are given in Table 2. An example of the outcome of the solubility tests are given as scores in Table 3. These scores, together with the scores of other solvents, were used to calculate the HSP values using the HSPiP program.

N2200 was easily dissolved in *o*-xylene in less than an hour and was dissolved in tetrahydronaphthalene in less than 24 hours. For mesitylene, however, 96 hours was required to dissolve N2200, even at a concentration as low as 1.0 mg/ml. The longer time required for this solvent can be understood in terms of the low values for δP and δH. In other words, more time is needed to establish the necessary polar interactions when an apolar solvent such as mesitylene is used. For toluene and ethyl benzene, there is undissolved N2200 even after 96 hours. For these solvents, it is probably the low value of δP that causes the mismatch between solute and solvent. One could argue that, according to the HSP values, toluene and ethyl benzene should work as well as mesitylene as a solvent for N2200. That is true, but emphasizes the fact that the HSP model relies on several assumptions and that solution chemistry is a very complex field. Finally, tetrahydrofuran was not able to solubilize N2200 partially, not even after 96 hours. In this case, it seems like the polar interactions are too strong to match N2200.

**Figure 4:**
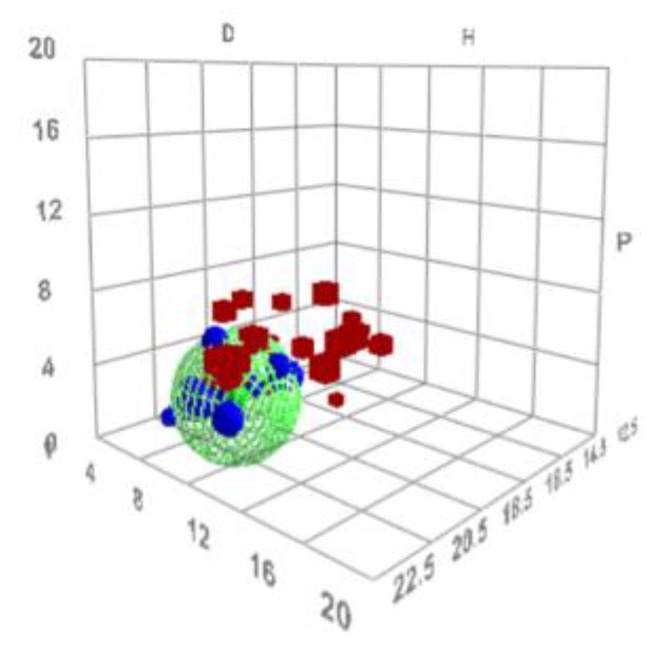
The solubility sphere for N2200 in Hansen space, as calculated by the HSPiP program. The centre of the sphere has the coordinates of the HSP-values of N2200. Red cubes denote bad solvents and blue spheres good solvents. The axes denoted D, P, and H denote δD, δP, and δH, respectively, in (MPa)^1/2^.

The calculations of the HSP values for N2200 resulted in δD = 18.4, δP = 0.7, δH = 2.3, and R_0_ = 3.9, and core values of ±[0.15, 0.50, 0.35]. From these values, it is possible to present the solubility of N2200 as a sphere in Hansen space, as seen in Figure 4.

**Figure 5:**
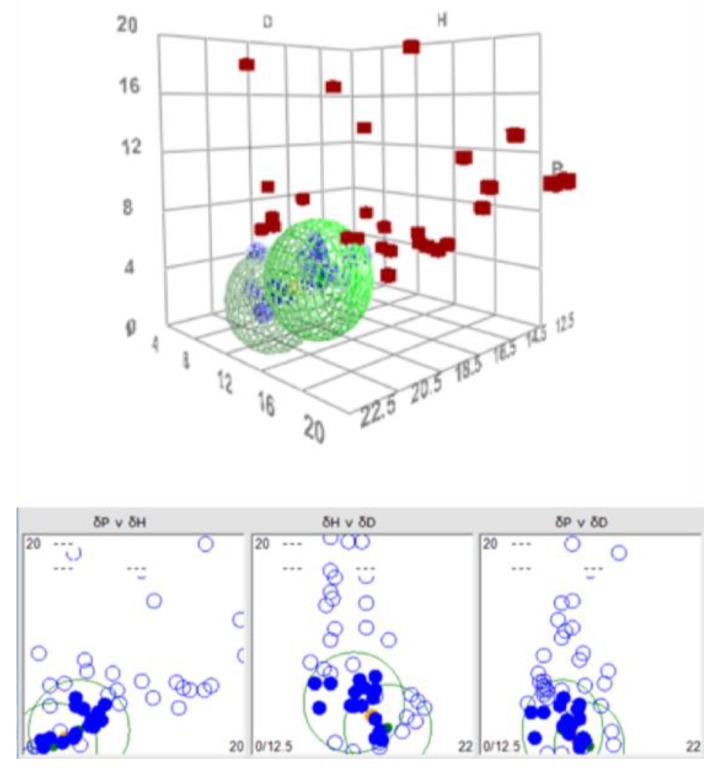
A double-sphere graphical representation of the solubilities of N2200 and TQ1. The region where the two spheres overlap, the junction, shows the part of Hansen space where it is likely to find solvents that work for both solutes. The junction is more easily seen in the two-dimensional projection below the three-dimensional Hansen space. The axes denoted D, P, and H denote δD, δP, and δH, respectively, in (MPa)^1/2^.

Using the HSP values for TQ1 determined by Holmes et al. [4], i.e., δD = 17.5, δP = 4.0, δH = 3.8, and R_0_ = 4.8, makes it possible to make a similar solubility sphere for this polymer. Putting these two spheres into the same three-dimensional graphical representation in Hansen space, as seen in Figure 5, shows an overlap region of the two spheres, the so-called junction. Sometimes, the junction is clearer in the two-dimensional projections, as shown in the lower part of Figure 5. Within this junction volume, it is likely that solvents that will be good for both solutes and thus serves as a good starting point when searching for solvent blends will be found. In this particular case, the center of the junction has the HSP values δD = 17.7, δP = 1.3, and δH = 3.4. This set of parameters was used as a target for modelling solvent blends for the pair TQ1:N2200.

It should be mentioned that the HSP values determined may differ from other HSP values given in the literature. In fact, every set of values are dependent on the actual experimental conditions and, hence, may differ. The general picture, however, will persist, in the sense that the relative weights of δD, δP, and δH will remain.

### 3.2. Alternative Solvent Blends for TQ1 and N2200

**Figure 6:**
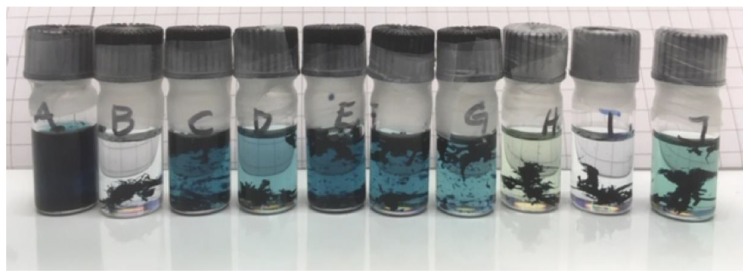
An example of the solubility tests of N2200 in the solvent blends calculated by the HSPiP program, at the initial stage of the test. The blends in the vials are (volume ratio in brackets); A: Toluene/1-methyl naphthalene (62/38), B: Cumene/benzaldehyde (72/28), C: Tetrahydronaphthalene/methyl acetate (88/12), D: Mesitylene/benzaldehyde (65/35), E: Tetrahydronaphthalene/o-xylene (65/35), F: Tetrahydronaphthalene/2-methyl tetrahydrofuran (77/23), G: Tetrahydronaphthalene/isobutyl acetate (85/15), H: Cumene/2-methyl anisole (60/40), I: Ethyl benzene/2-methyl anisole (53/47), J: Cumene/anisole (72/28).

With the target HSP of the junction between TQ1 and N2200, the HSPiP program can be used to model possible solvent blends. A set of ten solvent blends were found with promising HSP and RED values; see Table 4. An illustrative example of a solubility test is shown in Figure 6. The resulting scores of the solubility test are given in Table 4. The four blends being good solvents for N2200, i.e., A, C, E, and F, were tested as solvent blends for TQ1. All blends acted as good solvents, and it was possible to dissolve 10 mg/ml of TQ1 in each of the tested blends. This is also as expected, given the larger R_0_ for TQ1 as compared to the R_0_ of N2200. A larger R_0_ is indicative for a broader range of solvents to act as good solvents.

The shortest distance between N2200 and the solvent blend was found for blends F and G, followed by blend E, while the largest distance was found for blends I and J. Evidently, the R_a_ does not on its own give a precise picture of the solubility of N2200 in the blends—E and F are good solvents, while G is not as good. This is a demonstration of the fact that the HSP model relies on ideal mixing, which is not the case for all blends. This is also clear when comparing the RED values: All RED values are below 1, but not all blends are good solvents. One should remember that the HSP model assumes spherical solubility in Hansen space, which is not necessarily true. Consequently, one needs to perform the solubility tests to discriminate between the calculated solvent blends.

The remaining test was to dissolve the pair TQ1:N2200 in a weight ratio of 1:1 in the promising solvent blends. All four solvent blends turned out to dissolve the two solutes up to a total concentration of 10 mg/ml. It should be pointed out that some of the blends had a very high viscosity, even at 50 °C, making them less promising for applications. 

### 3.3. TQ1 and N2200 Film Morphology

**Figure 7:**
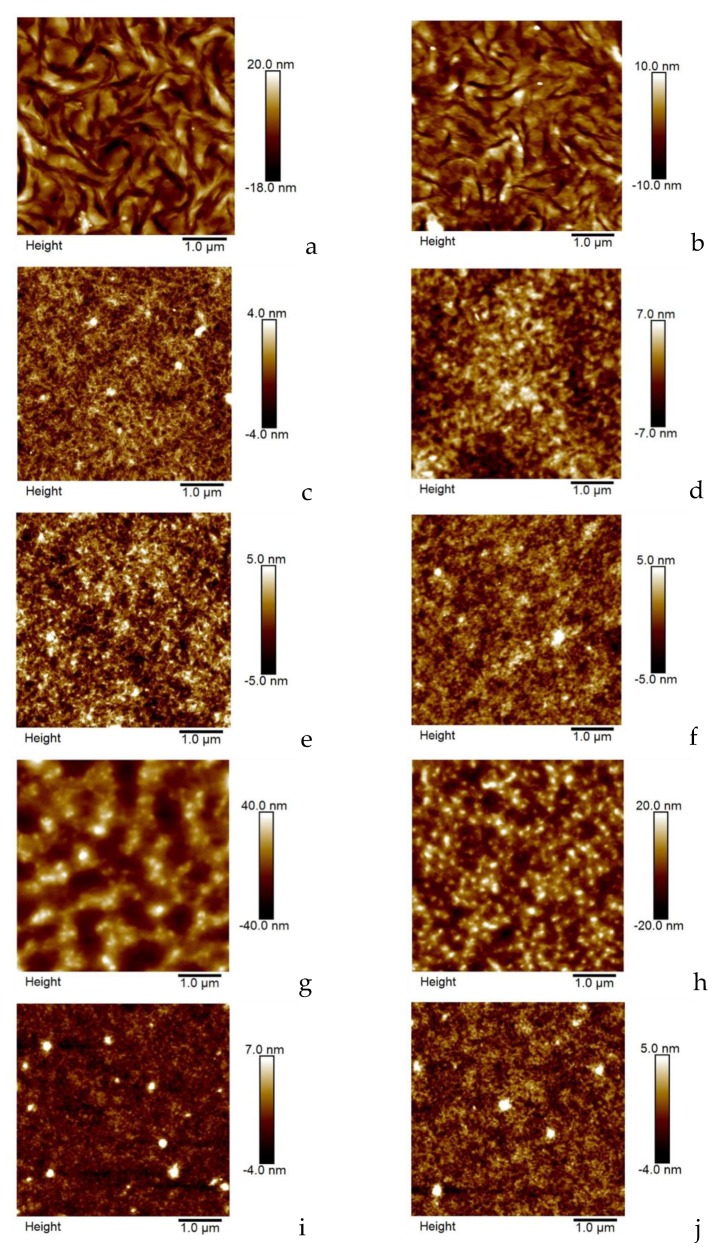
Atomic force microscopy (AFM) micrographs of blend films spun from various solvents. All films were spun at 1000 rpm and the total solute concentration was 10 mg/ml. All films, except a and b, were pre-heated at 250 °C for 1 minute to complete solvent evaporation. All films were thermally annealed at 120 °C for 10 minutes. The TQ1:N2200 weight ratio was 1:1 in films a, c, e, g, and i, while the ratio was 2:1 in the other films. The solvents are chloroform (**a** and **b**), solvent blend A (**c** and **d**), solvent blend C (**e** and **f**), solvent blend E (**g** and **h**), and solvent blend F (**i** and **j**).

Blend films, with the TQ1:N2200 weight ratio of either 1:1 or 2:1, were spun on glass substrates from the solvent blends A, C, E, and F, as well as from chloroform, a solvent known to be good for both TQ1 and N2200. The dried and thermally annealed films’ morphologies were characterized by AFM measurements; see Figure 7. The TQ1:N2200 blend films do not show strong phase separation in any case, even if there is slightly more structure found in the film spun from chloroform. The reason for this can be sought in several parameters. First, the viscosities of the solvent blends are remarkably higher than that of chloroform, strongly influencing the liquid film deposition on the glass substrate. Second, the films spun from blend solvents are all pre-heated at 250 °C for 1 minute before thermal annealing, while the film spun from chloroform was not pre-heated. This extra annealing, when some solvent is still present, most probably will have an influence on the morphology of the films from solvent blends.

Taking these differences into account, we conclude that the solvent blends, modelled by the HSPiP program, yields thin blend films with *similar* morphologies to what is found when the film is prepared from a solution with a frequently used solvent. This shows that the HSP model and the HSPiP is a suitable tool for a preliminary screening of solvent and solvent blend possibilities, saving much time for experimental work.

## 4. Conclusions

We have shown that the Hansen Solubility Parameters and the program *Hansen Solvent Parameters in Practice* can be used to successfully model possible solvent blends of two polymers, relevant for organic solar cells, i.e., the electron donor TQ1 and the electron acceptor N2200.

From solubility experiments, the HSP parameters δD, δP, δH, R_0_, R_a_, and RED could be determined for N2200. Together with reported parameters for TQ1, the solubility spheres’ junction could be determined and used for solvent blend modelling.

Less harmful solvents can be used to yield similar film morphologies as those obtained with more common, halogenated and/or aromatic, solvents.

In order to undertake a possible forthcoming investigation on solar cell devices produced from new solvent blends, more experimental work is needed to increase the understanding of the role of the solution chemistry in these systems. For instance, blends of three solvents remain to be modelled and investigated, and optical microscopy could be used to monitor the prepared films on a larger scale, i.e., 0.1–1 mm.

## Figures and Tables

**Table 1:** List of solvents.

Solvent	Manufacturer	Grade	CAS
1-Butanol	ACROS	99%	71-36-3
1-Methylnaphthalene	ACROS	97%	90-12-0
1-Octanol	Sigma–Aldrich	≥99.5%	111.87-5
2-Butanol	EMSURE	for analysis	78-92-2
2-Methyl Tetrahydrofuran	Sigma–Aldrich	>99.5%	96-47-9
2-Propanol (IPA)	VWR Chemicals	AnalaR	67-63-0
Acetone	VWR Chemicals	100%	67-64-1
Amyl Acetate	Sigma–Aldrich	99%	628-63-7
Benzaldehyde	Sigma–Aldrich	≥99.5	100-52-7
Chloroform	EMSURE	for analysis	67-66-3
Cyclohexane	Merck KGaA	≥99.5%	110-82-7
Cyclohexanone	Sigma–Aldrich	≥99%	108-94-1
Cyclopentyl Methyl Ether	Sigma–Aldrich	≥99%	5614-37-9
Dimethyl Formamide (DMF)	Sigma–Aldrich	≥99%	68-12-02
Dimethyl Sulfoxide (DMSO)	VWR Chemicals	99%	67-68-5
Dipropyl Amine	Sigma–Aldrich	99%	142-84-7
Dipropylene Glycol	Sigma–Aldrich	99%	25265-71-8
Ethanol	VWR Chemicals	96%	64-17-5
Ethyl Acetate	Sigma–Aldrich	≥99.5%	141-78-6
Ethyl Benzene	Janssen Chimica	AnalaR Normapur	100-41-4
Formamide	Sigma–Aldrich	≥99.5%	75-12-07
Glycerol	Sigma–Aldrich	≥99.5%	56-81-5
Isobutyl Acetate	Sigma–Aldrich	99%	110-19-0
Isopropyl Benzene (Cumene)	Sigma–Aldrich	98%	98-32-8
Mesitylene	ACROS	99% extra pure	108-67-8
Methyl Acetate	Merck KGaA	≥99%	79-20-9
Methyl Cyclohexane	ACROS	99% extra pure	108-87-2
n-Butyl Acetate	Sigma–Aldrich	≥99.5%	123-86-4
N-Methyl Formamide	Sigma–Aldrich	99%	123-39-7
*o*-Xylene	Alfa Aesar	99%	95-47-6
Propylene Carbonate	Sigma–Aldrich	99.7%	108-32-7
Propylene Glycol	ACROS	99%	57-55-6
Tetrahydrofuran (THF)	Sigma–Aldrich	≥99%	109-99-9
Tetrahydronaphthalene	Fischer Scientific	Lab. Reagent grade	119-64-2
Toluene	VWR Chemicals	AnalaR	108-88-3

**Table 2:** Solvents used for the solubility tests and their corresponding HSP values.

Solvent	δD [(MPa)^1/2^]	δP [(MPa)^1/2^]	δH [(MPa)^1/2^]
1-Butanol	16.0	5.7	15.8
1-Octanol	16.0	5.0	11.2
2-Butanol	15.8	5.7	14.5
2-Methyl Tetrahydrofuran	16.9	5.0	4.3
2-Propanol (IPA)	15.8	6.1	16.4
Acetone	15.5	10.4	7.0
Amyl Acetate	15.8	3.3	6.1
Cyclohexane	16.8	0.0	0.2
Cyclohexanone	17.8	8.4	5.1
Cyclopentyl Methyl Ether	16.7	4.3	4.3
Dimethyl Formamide (DMF)	17.4	13.7	11.3
Dimethyl Sulfoxide (DMSO)	18.4	16.4	10.2
Dipropyl Amine	15.3	1.4	4.1
Dipropylene Glycol	16.5	10.6	17.7
Ethanol	15.8	8.8	19.4
Ethyl Acetate	15.8	5.3	7.2
Ethyl Benzene	17.8	0.6	1.4
Formamide	17.2	26.2	19.0
Glycerol	17.4	11.3	27.2
Isobutyl Acetate	15.1	3.7	6.3
Isopropyl Benzene (Cumene)	18.1	1.2	1.2
Mesitylene	18.0	0.6	0.6
Methyl Acetate	15.5	7.2	7.6
Methyl Cyclohexane	16.0	0.0	1.0
n-Butyl Acetate	15.8	3.7	6.3
N-Methyl Formamide	17.4	18.8	15.9
*o*-Xylene	18.0	2.6	2.8
Propylene Carbonate	20.0	18.0	4.1
Propylene Glycol	16.8	10.4	21.3
Tetrahydrofuran (THF)	16.8	5.7	8.0
Tetrahydronaphthalene	19.6	2.0	2.9
Toluene	18.0	1.4	2.0

**Table 3:** Solubility scores for N2200, at a concentration of 1.0 mg/ml, in some solvents. Solvents that had a score of 0 in every test are not included in the table. δD, δP, and δH are given in (MPa)^1/2^.

Solvent	δD	δP	δH	Score 1 h	Score 24 h	Score 48 h	Score 72 h	Score 96 h
Ethyl Benzene	17.8	0.6	1.4	3	2	2	2	2
Mesitylene	18.0	0.6	0.6	3	2	2	2	1
*o*-Xylene	18.0	2.6	2.8	1	1	1	1	1
Tetrahydrofuran (THF)	16.8	5.7	8.0	4	3	3	3	3
Tetrahydronaphthalene	19.6	2.0	2.9	2	1	1	1	1
Toluene	18.0	1.4	2.0	2	2	2	2	2

**Table 4:** Solvent blends for N2200. The blend ratio is given as volume percentage. The solubility tests were performed for 24 hours at 50 °C. First, the lowest concentration was tested, and subsequently the concentration was increased for good solvent blends. δD, δP, and δH are given in (MPa)^1/2^.

Test #	Solvent Blend	V%	R_a_	RED	δD	δP	δH	Score	Score	Score
								mg/ml	mg/ml	mg/ml
								1.0	5.0	10.0
A	Toluene	62.0	1.2	0.69	18.6	1.3	2.0	1	1	1
	1-Methyl Naphthalene	38.0								
B	Isopropyl Benzene (Cumene)	72.0	1.1	0.64	18.5	2.9	2.3	3		
	Benzaldehyde	28.0								
C	Tetrahydronaphthalene	88.0	0.9	0.52	19.1	2.6	3.5	1	1	1
	Methyl Acetate	12.0								
D	Mesitylene	65.0	1.1	0.68	18.5	3.0	2.2	3		
	Benzaldehyde	35.0								
E	Tetrahydronaphthalene	65.0	0.7	0.42	19.0	2.7	3.2	1	1	1
	*o*-Xylene	35.0								
F	Tetrahydronaphthalene	77.0	0.6	0.36	18.4	2.6	2.0	1	1	1
	2-Methyl Tetrahydrofuran	23.0								
G	Tetrahydronaphthalene	85.0	0.6	0.34	18.9	2.3	3.4	2		
	Isobutyl Acetate	15.0								
H	Isopropyl Benzene (Cumene)	60.0	1.3	0.77	18.2	2.6	2.6	3		
	2-Methyl Anisole	40.0								
I	Ethyl Benzene	53.0	1.6	0.92	18.0	2.5	3.0	3		
	2-Methyl Anisole	47.0								
J	Isopropyl Benzene (Cumene)	72.0	1.6	0.93	18.0	2.1	2.8	2		
	Anisole	28.0								

## References

[1] Sharma A., Pan X., Bjuggren J.M., Gedefaw D., Xu X., Kroon R., Wang E., Campbell J.A., Lewis D.A., Andersson M.R. (2019). Probing the Relationship between Molecular structures, Thermal Transitions, and Morphology in Polymer Semiconductors Using a Woven Glass-Mesh-Based DMTA Technique. Chem. Mater..

[2] Zhu J., Liu Q., Li D., Xiao Z., Chen Y., Hua Y., Yang S., Ding L. (2019). A Wide-Band Gap Copolymer Donor for Efficient Fullerene-Free Solar Cells. ACS Omega.

[3] Uranbileg N., Gao C., Yang C., Bao X., Han L., Yang R. (2019). Amorphous electron donros with controllable morphology for non-fullerene polymer solar cells. J. Mater. Chem. C.

[4] Holmes N.P., Munday H., Barr M.G., Thomsen L., Marcus M.A., Kilcoyne A.L.D., Fahy A., van Stam J., Dastoor P.C., Moons E. (2019). Unravelling donor-acceptor film morphology formation for environmentally-friendly OPV ink formulations. Green Chem..

[5] Lindqvist C., Moons E., van Stam J. (2018). Fullerene Aggregation in Thin Films of Polymer Blends for Solar Cell Applications. Materials.

[6] Cheng P., Li G., Zhan X., Yang Y. (2018). Next-generation organic photovoltaics based on non-fullerene acceptors. Nat. Photonics.

[7] Cooling N.A., Barnes E.F., Almyahi F., Feron K., Al-Mudhafer M.F., Al-Ahmad A., Vaughan B., Andersen T.R., Griffith M.J., Hart A.S. (2016). A low-cost mixed fullerene acceptor blend for printed electronics. J. Mater. Chem. A.

[8] Blazinic V., Ericsson L.K.E., Muntean S.A., Moons E. (2018). Photo-degradation in air of spin-coated PC_60_BM and PC_70_BM films. Synth. Met..

[9] Morvillo P., Bobeico E., Esposito S., Diana R. (2012). Effect of the active layer thickness on the device performance of polymer solar cells having [60]PCBM and [70]PCBM as electron acceptor. Energy Procedia.

[10] Bruno A., Villani F., Grimaldi I.A., Loffredo F., Morvillo P., Diana R., Haque S., Minarini C. (2014). Morphological and spectroscopic characterizations of inkjet-printed poly(3-hexylthiophene-2,5-diyl): Phenyl-C61-butyric acid methyl ester blends for organic solar cell applications. Thin Solid Film..

[11] Morvillo P., Ricciardi R., Nenna G., Bobeico E., Diana R., Minarini C. (2016). Elucidating the origin of the improved current output in inverted polymer solar cells. Sol. Energy Mater. Sol. Cells.

[12] Lee H., Park C., Sin D.H., Park J.H., Cho K. (2018). Recent Advances in Morphology Optimization for Organic Photovoltaics. Adv. Mater..

[13] Hansen C.M. (2000). Hansen Solubility Parameters. A User’s Handbook.

[14] Machui F., Brabec C.J., Yang X. (2012). Solubility, Miscibility, and the Impact on Solid-State Morphology. Semiconducting Polymer Composites: Principles, Morphologies, Properties and Applications.

[15] Miller-Chou B.A., Koenig J.L. (2003). A review of polymer dissolution. Prog. Polym. Sci..

[16] Agata Y., Yamamoto H. (2018). Determination of Hansen solubility parameters of ionic liquids using double-sphere type of Hansen solubility sphere method. Chem. Phys..

[17] Zhu Q.-N., Wang Q., Hu Y.-B., Abliz X. (2019). Practical Determination of the Solubility Parameters of 1-Alkyl-3-methylimidazolium Bromide ([CnC1im]Br, n = 5, 6, 7, 8) Ionic Liquids by Inverse Gas Chromatography and the Hansen Solubility Parameter. Molecules.

[18] Park C.-D., Fleetham T.A., Li J., Vogt B.D. (2011). High performance bulk-heterojunction organic solar cells fabricated with non-halogenated solvent processing. Org. Electron..

[19] Duong D.T., Walker B., Lin J., Kim C., Love J., Purushothaman B., Anthony J.E., Nguyen T.-C. (2012). Molecular Solubility and Hansen Solubility Parameters for the Analysis of Phase Separation in Bulk Heterojunctions. J. Polym. Sci. B Polym. Phys..

[20] Burgués-Ceballos I., Machui F., Min J., Ameri T., Voigt M.M., Luponosov Y.N., Ponomarenko S.A., Lacharmoise P.D., Campoy-Quiles M., Brabec C.J. (2014). Solubility Based Identification of Green Solvents for Small Molecule Organic Solar Cells. Adv. Funct. Mater..

[21] Machui F., Langner S., Zhu X., Abbott S., Brabec C.J. (2012). Determination of the P3HT:PCBM solubility parameters via a binary solvent gradient method: Impact of the solubility on the photovoltaic performance. Sol. Energy Mater. Sol. Cells.

[22] Hansen Solubility Parameters in Practice. https://www.hansen-solubility.com/contact.php.

[23] Royal Society of Chemistry GSK Solvent Selection Guie 2009. http://www.rsc.org/suppdata/gc/c0/c0gc00918k/c0gc00918k.pdf.

